# Acute Scrotal Ulcers in a 34-Year-Old Male With an Excellent Treatment Response: A Case Report

**DOI:** 10.7759/cureus.92952

**Published:** 2025-09-22

**Authors:** João N Soares, Maria M Brites, Jose C Cardoso

**Affiliations:** 1 Dermatology, Coimbra University Hospital, Coimbra, PRT; 2 Faculty of Medicine, University of Coimbra, Coimbra, PRT

**Keywords:** epstein-barr virus, genital ulcers, rare skin disease, scrotal ulcer, vasculitis

## Abstract

Juvenile gangrenous vasculitis of the scrotum (JGVS) is a rare disease, but it has a distinct presentation and a favourable response to treatment. In this clinical case, we present a male patient in his early 30s with tender scrotal ulcers, no history of sexually transmitted diseases or risk behaviours, and no previous systemic disease or immunosuppression. Studies revealed elevated inflammatory markers, but remarkably, all microbiologic studies were negative. The patient was diagnosed with JGVS and was treated with methylprednisolone 24 mg, tapered over two months, in combination with doxycycline 100 mg BID for one month, and topical clobetasol under occlusion, with complete healing in two months and no relapse. This case contributes to the existing knowledge of JGVS. Typically, patients are young, usually under 30 years old; however, this patient was slightly older. While exclusive scrotal involvement is common, this patient also exhibited smaller satellite ulcers on the adjacent thigh.

## Introduction

Juvenile gangrenous vasculitis of the scrotum (JGVS) is an uncommon, self-limiting ulcerative disease of the male genitalia, first described by Piñol Aguadé in 1973 in a case series of five young men from Barcelona [1,2]. Since then, a few cases have been reported worldwide [2-5].

The pathogenesis remains poorly understood. Several reports have noted a prodrome of flu-like symptoms preceding ulcer development, raising the possibility of a post-infectious or immune-mediated mechanism, with Epstein-Barr virus (EBV) infection being the most frequently implicated trigger [5,6]. Histologically, JGVS shows a dense neutrophilic infiltrate and fibrinoid necrosis of small vessels, overlapping with features of neutrophilic dermatoses but without evidence of microorganisms [2]. This has led to debate as to whether JGVS should be considered a localized form of pyoderma gangrenosum (PG) or a male counterpart of Lipschütz ulcer in females [6-8].

Clinically, JGVS typically presents in adolescents or young adults under 30 years of age as the abrupt onset of painful, well-demarcated scrotal ulcers, often accompanied by fever and systemic inflammation. The ulcers usually resolve within four to eight weeks following treatment with systemic corticosteroids and/or antibiotics, without recurrence [3,9]. Recognition is important because JGVS can mimic sexually transmitted infections, Behçet disease, or necrotizing infections, which may lead to unnecessary antimicrobial therapy or invasive procedures.

In this report, we present the case of a 34-year-old man with scrotal and satellite thigh ulcers, representing an unusual presentation of JGVS due to the slightly older age of onset and the atypical distribution of lesions. This case contributes to the expanding spectrum of clinical features and reinforces the need for awareness of this rare but distinctive condition.

## Case presentation

**Figure 1:**
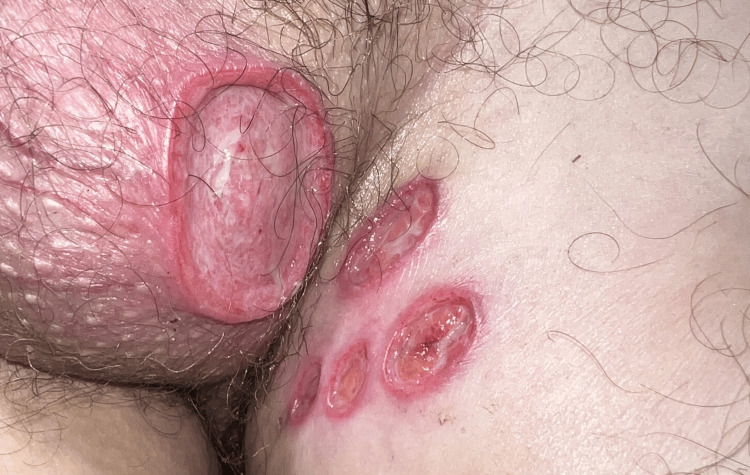
Five oval ulcers of varying sizes (0.5-4 cm), distributed over the left hemiscrotum and medial upper left thigh in a “kissing pattern,” where the adjacent surfaces were affected symmetrically.

A male patient in his 30s presented to the Emergency Department due to scrotal ulcers that had progressively worsened over three weeks. Prior to the onset, he described having flu-like symptoms. Physical examination revealed five oval ulcers of varying sizes (0.5-4 cm), distributed over the left hemiscrotum and medial upper left thigh in a “kissing pattern,” where the adjacent surfaces were affected symmetrically (Figure 1). The borders were sharply demarcated, with a pseudo-undermining appearance. The lesions were tender, and there was no inguinal lymphadenopathy or oral ulcers. Palpation of the lesions revealed they were superficial, mobile, and lacked any underlying masses or nodules. There was no skin retraction or signs of deep tissue extension. The patient had no systemic underlying condition or immunosuppression, denied any previous sexually transmitted disease or risk behaviour, and had no regular medication.

From the patient's perspective, enduring the progressive worsening of scrotal ulcers over three weeks, without a clear diagnosis or underlying condition, evoked feelings of anxiety and discomfort, compounded by the physical pain and uncertainty regarding the origin of his symptoms.

**Figure 2:**
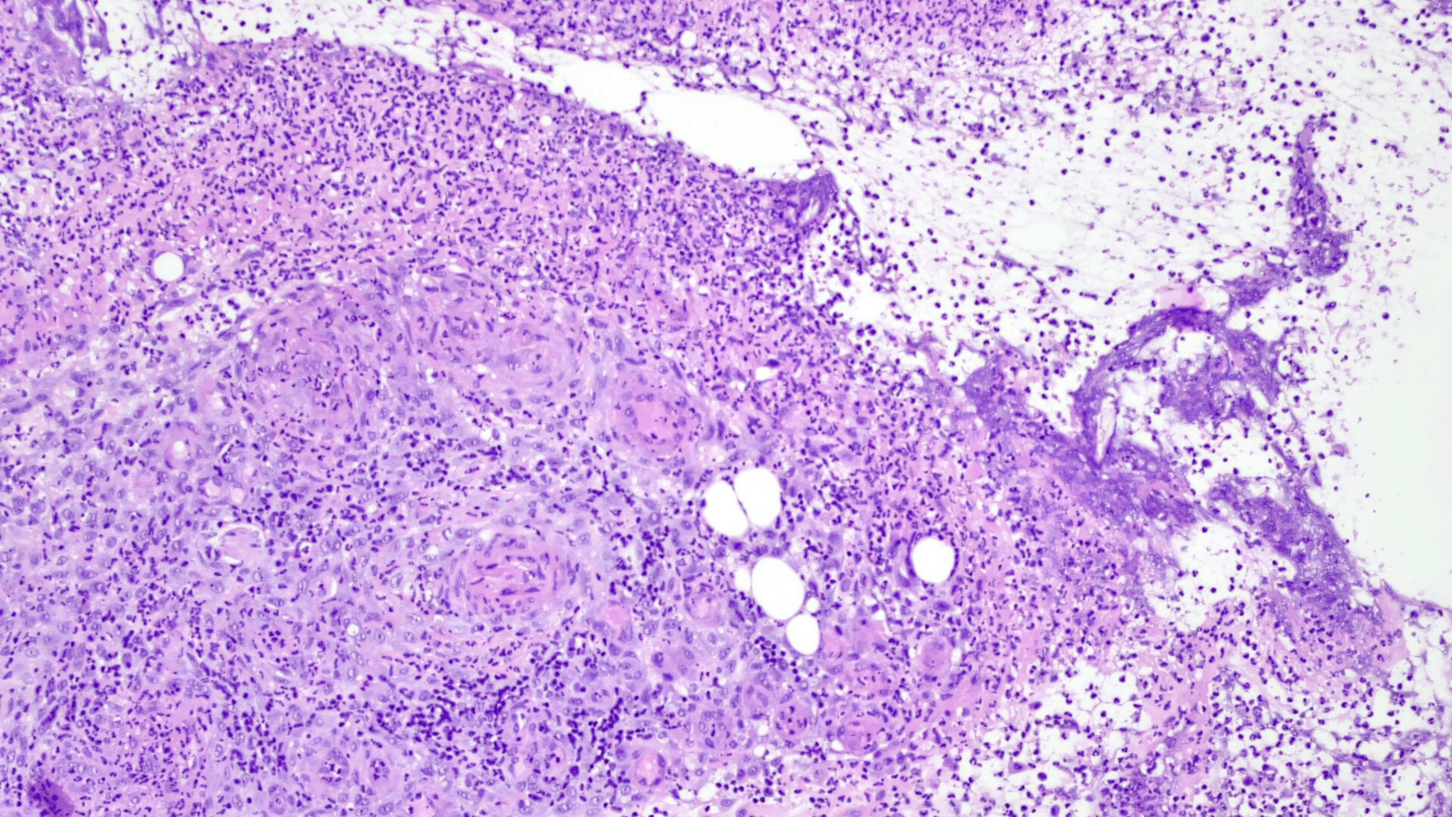
Biopsy of the scrotal ulcer displaying a dense neutrophilic infiltrate associated with secondary fibrinoid necrosis of small blood vessels (haematoxylin and eosin, ×200).

Relevant laboratory results are included in Table 1. Biopsy of the scrotal ulcer revealed a dense superficial and deep inflammatory infiltrate, composed predominantly of neutrophils, associated with secondary fibrinoid necrosis of small blood vessels (Figure 2). No microorganisms were identified with Gram, Epstein-Barr virus-encoded RNA (EBER), or cytomegalovirus (CMV) staining, or on culture.

**Table 1:** Relevant laboratory test results showing elevation of inflammatory markers, anaemia, and thrombocytosis attributable to inflammation. Notably, all microbiologic studies were negative except for EBV, compatible with past infection. HBV: hepatitis B virus; HCV: hepatitis C virus; HIV: human immunodeficiency virus; HSV: herpes simplex virus; CMV: cytomegalovirus; EBV: Epstein-Barr virus; PCR: polymerase chain reaction

Lab Test	Value	Normal Range
C-Reactive Protein	21.54 mg/dL	< 0.5 mg/dL
Leukocytes	23 x 10^9^/L	3.9-10.2 x 10^9^/L
Neutrophils	20.26 x 10^9^/L	1.5-7.7 x 10^9^/L
Haemoglobin	11.8 g/dL	13.5-17.5 g/dL
Platelets	708 x 10^9^/L	150-450 x 10^9^/L
HBV serology	Negative	
HCV serology		
HIV serology		
Syphilis screening		
Interferon gamma assay		
EBV serology	IgG positive (42.8) and IgM negative (0.3) 3 months after disease onset	
HSV-1 and 2 lesion swab PCR	Negative	

Differential diagnosis

The differential diagnosis of acute scrotal and genital ulcers is broad. In this case, the main considerations included JGVS, PG, infectious genital ulcers (herpes simplex virus, chancroid, EBV-associated ulcers, and mpox), and Behçet disease. The lesions shared some morphological overlap with PG [5], particularly the pseudo-undermined borders. However, histopathology revealed neutrophilic vasculitis with fibrinoid necrosis of small vessels, which is less typical of PG. Furthermore, the 2018 Delphi consensus diagnostic criteria for PG [10] were not met in this patient: no pathergy phenomenon, no systemic disease association, and the disease course was self-limited with full remission in two months. These findings strongly support JGVS over PG. Among infectious aetiologies, ulcerative genital herpes and chancroid were excluded by negative microbiological testing and absence of risk behaviours. Mpox was also considered, given the recent outbreaks; however, the patient lacked systemic features or lymphadenopathy, and although orthopoxvirus polymerase chain reaction (PCR) testing was not performed, the course and healing pattern were inconsistent with mpox. Behçet disease was excluded given the absence of oral ulcers, ocular disease, arthritis, or other systemic manifestations.

Treatment

Given the uncertain diagnosis, the patient was initially treated with doxycycline 100 mg every 12 hours for one month and topical clobetasol 0.5 mg/g mixed with fucidic acid 20 mg/g BID under occlusion, but there was little improvement. After tests ruled out infection as the cause of the ulcer, the patient was started on 24 mg methylprednisolone, tapered over two months (24 mg for two weeks, 16 mg for two weeks, 8 mg for two weeks, 4 mg for two weeks, then stopped).

Outcome and follow-up

**Figure 3:**
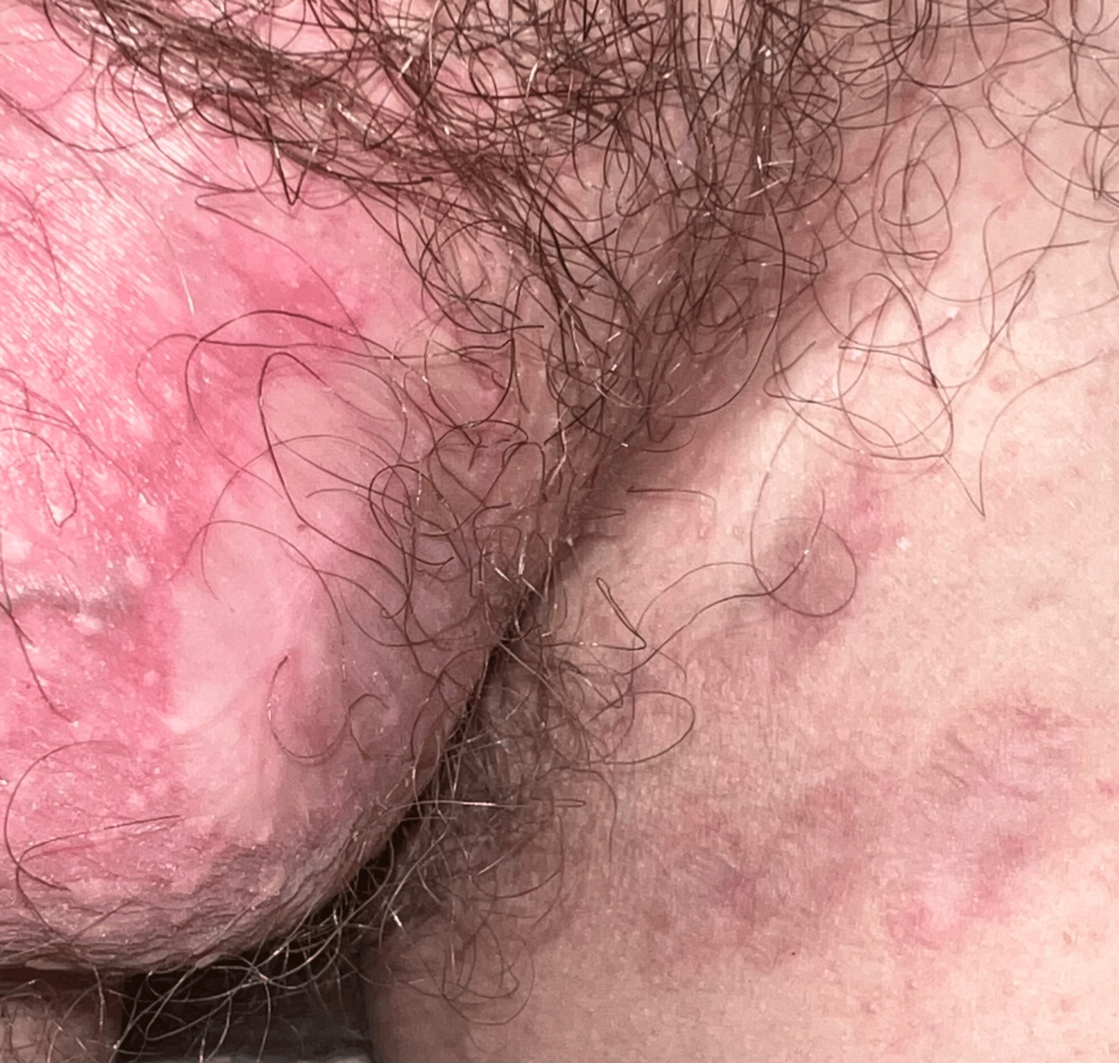
Resolution of the ulcers, with post-inflammatory scarring, observed after two months of treatment.

The lesions healed after two months, with post-inflammatory scarring (Figure 3). He was able to return to daily activities and work without limitations. The patient remains without recurrence after 20 months of follow-up.

## Discussion

This report describes JGVS in a 34-year-old man with satellite thigh lesions, an unusual phenotype that broadens the recognized clinical spectrum of this rare disease. While most published cases involve men under 30 years of age with ulcers confined to the scrotum, this case highlights that an older age of onset and extragenital extension can also occur.

These atypical features reinforce the need for clinicians to maintain a high index of suspicion when evaluating genital ulcers with an abrupt onset and negative microbiologic studies. In particular, thigh involvement could easily lead to misclassification as PG or other neutrophilic dermatoses [10,11]. Our findings suggest that JGVS may not be as strictly age-restricted or site-limited as previously assumed, highlighting the importance of careful clinicopathologic correlation rather than reliance on rigid diagnostic expectations.

Recently, SARS-CoV-2 has been reported as a possible immune-mediated trigger for acute genital ulcers in both males and females, particularly when preceded by flu-like symptoms. Although we did not test for SARS-CoV-2 in this case, such testing may be considered in similar scenarios, depending on the epidemiological context, without implying causality, but acknowledging a potential hypothesis-generating association.

Another important implication concerns the therapeutic response. Similar to previous reports, our patient experienced complete healing within two months after treatment with systemic corticosteroids and doxycycline, with no recurrence during one year of follow-up. This outcome underscores the self-limited nature of the disease and the consistency of corticosteroid responsiveness across different clinical presentations. Early recognition is, therefore, critical to avoid unnecessary antimicrobial regimens or invasive interventions and to initiate effective therapy promptly.

## Conclusions

JGVS is a rare, self-limited condition that should be considered in the differential diagnosis of acute genital ulcers, particularly when microbiologic studies are negative and histology shows neutrophilic vasculitis without microorganisms. Prompt recognition is essential to prevent unnecessary antimicrobial therapy or invasive interventions and to initiate corticosteroid treatment, which consistently results in rapid healing and excellent prognosis.

This case illustrates an uncommon presentation with satellite thigh lesions, reinforcing the clinical variability of JGVS, and the need for awareness among clinicians to ensure timely diagnosis and appropriate management.
